# Retraction: Ascertaining the Prevalence of Group B Streptococcal Infection in Patients with Preterm Premature Rupture of Membranes: A Cross-Sectional Analysis from Pakistan

**DOI:** 10.7759/cureus.r28

**Published:** 2021-03-05

**Authors:** Saiqa Yaseen, Shumaila Asghar, Irum Shahzadi, Abdul Qayyum

**Affiliations:** 1 Obstetrics and Gynaecology, Lady Willingdon Hospital, Lahore, PAK; 2 Obstetrics and Gynaecology, Sir Ganga Ram Hospital, Lahore, PAK; 3 Pediatric Surgery, Fatima Memorial Hospital, Lahore, PAK

This article has been retracted due to allegations that the study data was plagiarized from an already-published article in Annals of Punjab Medical College:​​​​ http://apmcfmu.com/index.php/apmc/article/download/796/605

The authors of this article as well as their Department Chair were all contacted regarding these allegations and provided with ample opportunity to respond. As no response has been received, we are retracting this article due to likely plagiarism of the above-linked study.

